# Inhibitory effect of anti-malarial agents on the expression of proinflammatory chemokines via Toll-like receptor 3 signaling in human glomerular endothelial cells

**DOI:** 10.1080/0886022X.2021.1908901

**Published:** 2021-04-05

**Authors:** Riko Sato, Tadaatsu Imaizumi, Tomomi Aizawa, Shojiro Watanabe, Koji Tsugawa, Shogo Kawaguchi, Kazuhiko Seya, Tomoh Matsumiya, Hiroshi Tanaka

**Affiliations:** a Department of Pediatrics, Hirosaki University Hospital, Hirosaki, Japan; b Department of Vascular Biology, Hirosaki University Graduate School of Medicine, Hirosaki, Japan; c Faculty of Education, Department of School Health Science, Hirosaki University, Hirosaki, Japan

**Keywords:** Chloroquine, glomerular endothelial cells, hydroxychloroquine, lupus nephritis, Toll-like receptor 3

## Abstract

**Objective:**

Although anti-malarial agents, chloroquine (CQ) and hydroxychloroquine (HCQ) are currently used for the treatment of systemic lupus erythematosus, their efficacy for lupus nephritis (LN) remains unclear. Given that upregulation of glomerular Toll-like receptor 3 (TLR3) signaling plays a pivotal role in the pathogenesis of LN, we examined whether CQ and HCQ affect the expression of the TLR3 signaling-induced representative proinflammatory chemokines, monocyte chemoattractant protein-1 (MCP-1), and C–C motif chemokine ligand 5 (CCL5) in cultured human glomerular endothelial cells (GECs).

**Methods:**

We examined the effect of polyinosinic-polycytidylic acid (poly IC), an agonist of TLR3, on MCP-1, CCL5 and interferon (IFN)-β expression in GECs. We then analyzed whether pretreatment with CQ, HCQ, or dexamethasone (DEX) inhibits poly IC-induced expression of these chemokines using real-time quantitative reverse transcriptase PCR and ELISA. Phosphorylation of signal transducers and activator of transcription protein 1 (STAT1) was examined using western blotting.

**Results:**

Poly IC increased MCP-1 and CCL5 expression in a time- and concentration-dependent manner in GECs. Pretreating cells with CQ, but not DEX, attenuated poly IC-induced MCP-1 and CCL5 expression; however, HCQ pretreatment attenuated poly IC-induced CCL5, but not MCP-1. HCQ did not affect the expression of IFN-β and phosphorylation of STAT-1.

**Conclusion:**

Considering that TLR3 signaling is implicated, at least in part, in LN pathogenesis, our results suggest that anti-malarial agents exert a protective effect against the development of inflammation in GECs, as postulated in LN. Interestingly, CQ is a rather powerful inhibitor compared with HCQ on TLR3 signaling-induced chemokine expression in GECs. In turn, these findings may further support the theory that the use of HCQ is safer than CQ in a clinical setting. However, further detailed studies are needed to confirm our preliminary findings.

## Introduction

Given that viral infections may trigger either the development of inflammatory renal disease or the worsening of preexisting renal disease [1], activated signaling through Toll-like receptor 3 (TLR3) reportedly plays a crucial role in the pathogenesis of glomerulonephritis (GN) [2,3]. Concerning TLR3 in resident glomerular cells, both exogenous ligands derived from pathogens and endogenous ligands can activate TLR3 and downstream immune responses leading to the development of ‘pseudo’ antiviral immunity-related inflammations in the kidney [3–5]. The activation of these TLRs, including TLR3, signaling cascade in residual renal cells and cross-talk of infiltrating monocytes, neutrophils, and lymphocytes result in inducing type I interferon (IFN) release, which may be involvement of pathogenesis of lupus nephritis (LN) [3–5]. Further, the implication of continuous activation of type I IFN system has been reported to play a pivotal role in the pathogenesis of systemic lupus erythematosus (SLE) [6]. Therefore, this theory is probably involved in the pathophysiology of GN, especially in LN [3–6]. Expression of TLR3 in resident glomerular cells has been confirmed in biopsy specimens from patients with LN [7]. Among these cells, glomerular endothelial cells (GECs) are directly exposed to circulating viral particles in the glomerulus [8,9]. Thus, the specific molecular mechanisms underlying the initiation of glomerular inflammation through the activation of endothelial TLR3 signaling need to be determined. Thus far, we found that endothelial TLR3 activation leads to inflammatory chemokine and adhesion molecule expression in cultured human GECs [8–12]. Despite some limitations, endothelial TLR3 signaling, which is associated with the continuous activation of type I interferon (IFN) as well as the regional expression of various inflammatory molecules in GECs, is thought to be involved in the pathogenesis of LN [3,6].

Although the European League against Rheumatism and the European Renal Association-European Dialysis and Transplant Association (EULAR/ERA-EDTA) has recommended the use of the anti-malarial agents, chloroquine (CQ) and hydroxychloroquine (HCQ) for patients with systemic lupus erythematosus (SLE) and LN [13], the beneficial effects of these agents against glomerular inflammation in LN has not been elucidated yet. Notably, CQ and HCQ have been reported to directly interacts with nucleic acids and consequently cause structural modifications of the TLR ligand and prevent nucleic acids from binding to TLR, which inhibits TLR3 and TLR9 signaling [14]. Previously, we examined the effects of CQ on the expression of C–C motif ligand 5 (CCL5) via TLR3 signaling in human glomerular mesangial cells (MCs) and found that CQ attenuates mesangial TLR3 signaling in the early phase [15]. Intracellular signaling systems are sometimes different between different cell types, and we think it is important to examine the effect of antimalarial agents on GECs, another type of resident glomerular cell.

In the clinical setting, the occurrence of retinopathy, a serious adverse event of anti-malarial agents, is a major concern. Notably, the incidence of HCQ retinopathy is less likely than CQ retinopathy, suggesting that different modes of action between these drugs exist in the retinal cells [16]. However, it remains unclear whether such differences exist between CQ and HCQ in their inhibitory effects on the expression of TLR3 signaling-mediated proinflammatory functional molecules in resident glomerular cells. In this study, we examined whether CQ and HCQ differentially affect the expression of TLR3 signaling-mediated representative proinflammatory chemokines, monocyte chemoattractant protein-1 (MCP-1), and CCL5 in GECs.

## Methods

### Reagents

Poly IC was obtained from Sigma (St. Louis, MO, USA). Chloroquine diphosphate (CQ), hydroxychloroquine (HCQ), and dexamethasone (DEX) were purchased from Wako Pure Chemical Industries, Ltd (Osaka, Japan). Small-interfering RNAs (siRNAs) against IFN-β and Lipofectamine RNAi MAX were purchased from Invitrogen (Frederick, MD, USA), and siRNAs against NF-κB p65 were from Cell Signaling Technologies (Danvers, MA, USA). SsoAdvanced Universal SYBR Green Supermix was obtained from Bio-Rad (Hercules, CA, USA). Oligonucleotide primers for PCR were custom synthesized by FASMAC (Atsugi, Japan). Enzyme-linked immunosorbent assay (ELISA) kits for human CCL2/MCP-1 and human CCL5/RANTES were purchased from R&D Systems (Minneapolis, MN, USA). An ELISA kit for IFN-β was purchased from PBL Assay Science (Piscataway, NJ, USA). Anti-signal transducer and activator transcription 1 (STAT1) rabbit IgG (sc-592) and anti-phosphorylated STAT1 (p-STAT1) mouse IgG (sc-136229) were from Santa Cruz Biotechnology (Dallas, TX, USA).

### Cells

GECs were purchased from ScienCell (Carlsbad, CA, USA) and were cultured in endothelial growth medium-2 (EGM-2; Lonza, Walkersville, MD). The culture medium was supplemented with 5% fetal bovine serum, 50 μg/mL gentamicin, and 50 μg/mL amphotericin B. Poly IC was dissolved in phosphate-buffered saline (PBS), pH 7.4, and the cells were treated with 0.5–50 μg/mL poly IC for up to 24 h [8–11]. In the experiments using immunosuppressive reagents, GECs were pretreated with 1 or 10 μg/mL CQ, 1 or 10 μg/mL HCQ, or 10 µM DEX for 1 h before the treatment with 30 μg/mL poly IC. In our previous studies, we found that the cell viability was more than 95% when the cells were pretreated with up to 20 μg/mL CQ and HCQ. RNA interference experiments were performed using a specific siRNA against IFN-β (12), NF-κB p65, or non-silencing negative control siRNA using the Lipofectamine RNAi MAX.

### Real-time quantitative reverse transcription (RT) PCR analysis

Total RNA was extracted from cells using illustra RNA spin kit (GE healthcare, Buckinghamshire, UK). Single-stranded complementary DNA was synthesized from 1 μg of total RNA using oligo (dT)_18_ primers and Moloney murine leukemia virus reverse transcriptase (MMLVRT). The complementary DNA for MCP-1, CCL5, IFN-β , and glyceraldehyde-3-phosphate dehydrogenase (GAPDH) was amplified using SsoAdvanced Universal SYBR Green Supermix. All values were normalized to GAPDH mRNA levels. PCR was performed using the following primers:

MCP-1-F; 5′-AAACTGAAGCTCGCACTCTCGC-3′,

MCP-1-R; 5′-ATTCTTGGGTTGTTGAGTGAGT-3′,

CCL5-F; 5′-CTACTCGGGAGGCTAAGGCAGGAA-3′,

CCL5-R; 5′-GAGGGGTTGAGACGGCGGAAGC-3′,

IFN-β-F; 5′-ACTGCCTCAAGGAGAGGATG-3′,

IFN-β-R; 5′-AGCCAGGAGGTTCTCAACAA-3′,

GAPDH-F; 5′-GCACCGTCAAGGCTGAGAAC-3′, and

GAPDH-R; 5′-ATGGTGGTGAAGACGCCAGT-3′.

### ELISA for MCP-1, CCL5, and IFN-β protein

The concentrations of MCP-1, CCL5, and IFN-β proteins in the cell-conditioned medium were measured in triplicate using an ELISA kit according to the manufacturer’s protocol.

### Western blotting

The cells were lysed in Laemmli’s buffer after incubation, and the lysates were subjected to 5–20% polyacrylamide gel electrophoreses. The proteins were transferred to polyvinylidene difluoride membranes. After blocking, the membranes were probed with antibodies against STAT1 (1:10 000) or p-STAT1 (1:5000). The bands were visualized using horseradish peroxidase-labeled secondary antibodies and a chemiluminescent substrate.

### Statistical analysis

All experiments were performed at least three times. Values are reported as the means ± standard deviation (SD). Significance in differences between groups was analyzed using Student’s *t*-test. A *p*-value of less than 0.05 was considered statistically significant. All analyses were carried out using GraphPad Prism software version 7 (GraphPad Software, Inc., La Jolla, CA, USA).

## Results

### Poly IC induced the expression of MCP-1, CCL5, and IFN-β in cultured human GECs

**Figure 1.**
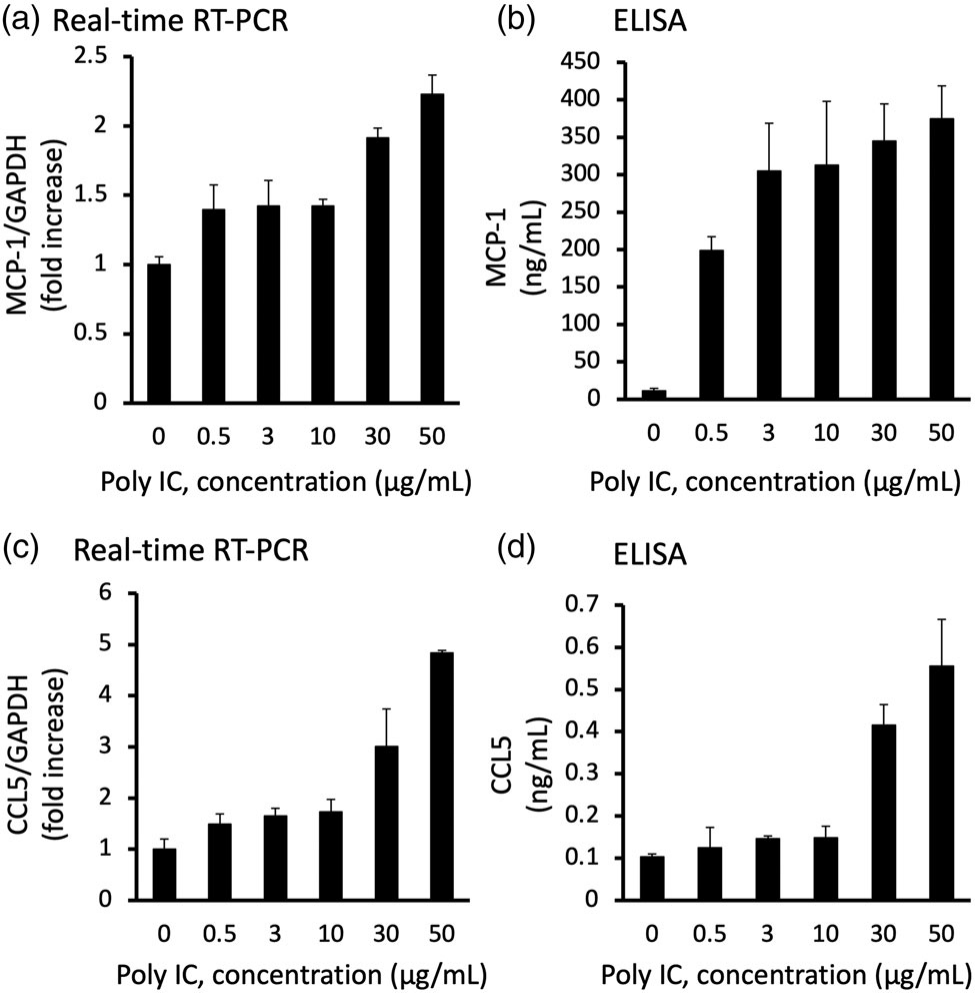
Poly IC induces the expression of MCP-1 and CCL5 in cultured human GECs in a concentration-dependent manner. The cells were treated with various concentrations of poly IC. After 24 h incubation, RNA was extracted and subjected to real-time RT-PCR (a,c). The concentrations of MCP-1 and CCL5 in the medium were analyzed using ELISA (b,d). Data are shown as the means ± SD (*n* = 3, *p < 0.01, by *t*-test).

**Figure 2.**
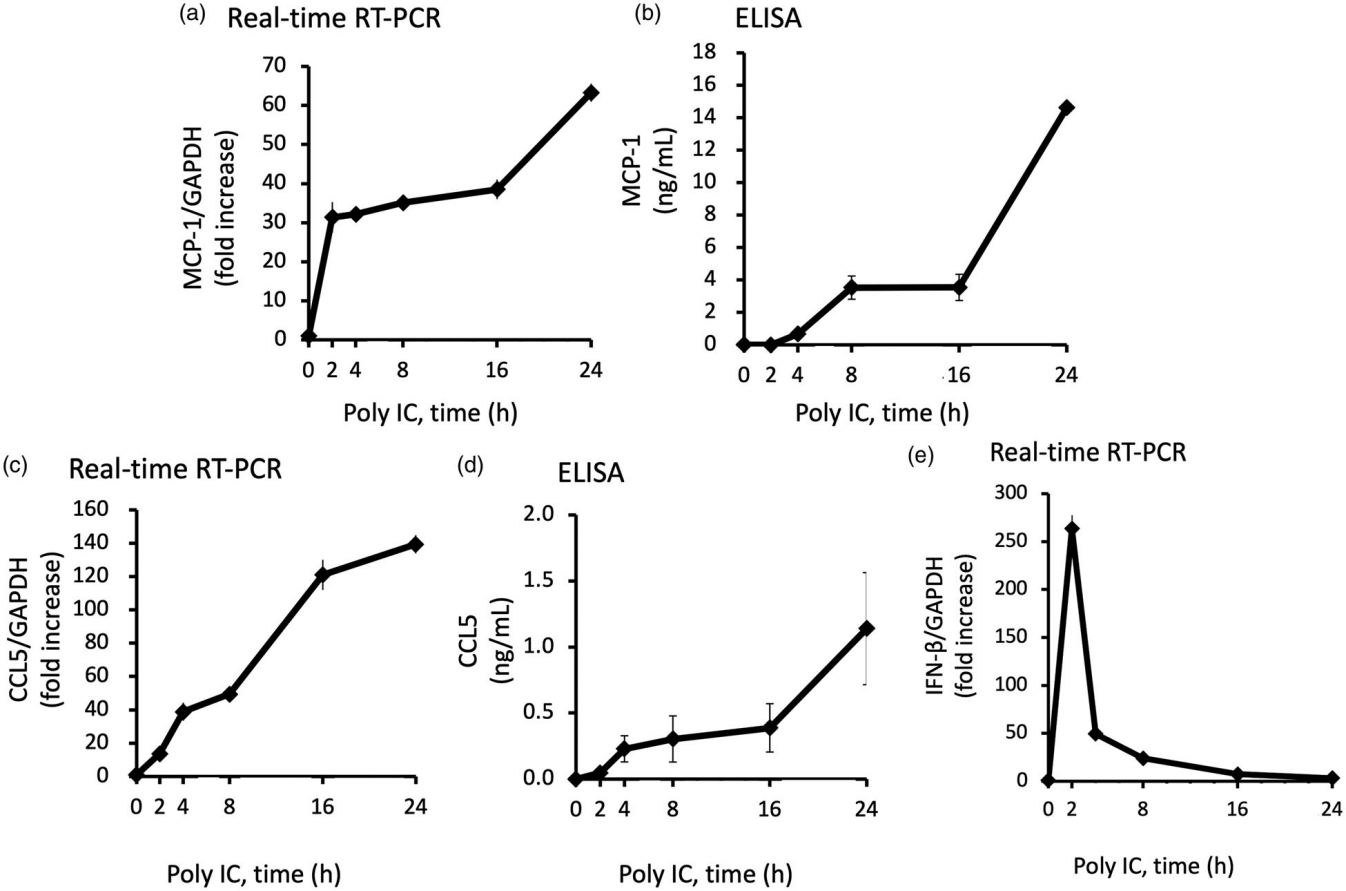
Poly IC induces the expression of MCP-1 and CCL5 in cultured human GECs in a time-dependent manner. The cells were treated with 30 μg/mL poly IC for up to 24 h. The conditioned medium was collected, and RNA was extracted from the cells. Real-time RT-PCR (a,c,e) and ELISA (b,d) analyses were performed. Data are shown as the means ± SD (*n* = 3, *p < 0.01, by *t*-test).

We confirmed that stimulation of GECs with poly IC resulted in increased expression of MCP-1 and CCL5 both at the mRNA and protein levels in a concentration- and time-dependent manner (Figures 1 and 2(a–d)). The expression level of MCP-1 and CCL5 mRNA increased gradually up to 24 h (Figure 2(a,c)). On the other hand, IFN-β mRNA peaked at 2 h and then decreased rapidly thereafter (Figure 2(e)).

### Knockdown of IFN-β or NF-κB p65 decreases the poly IC-induced expression of MCP-1, CCL5

**Figure 3.**
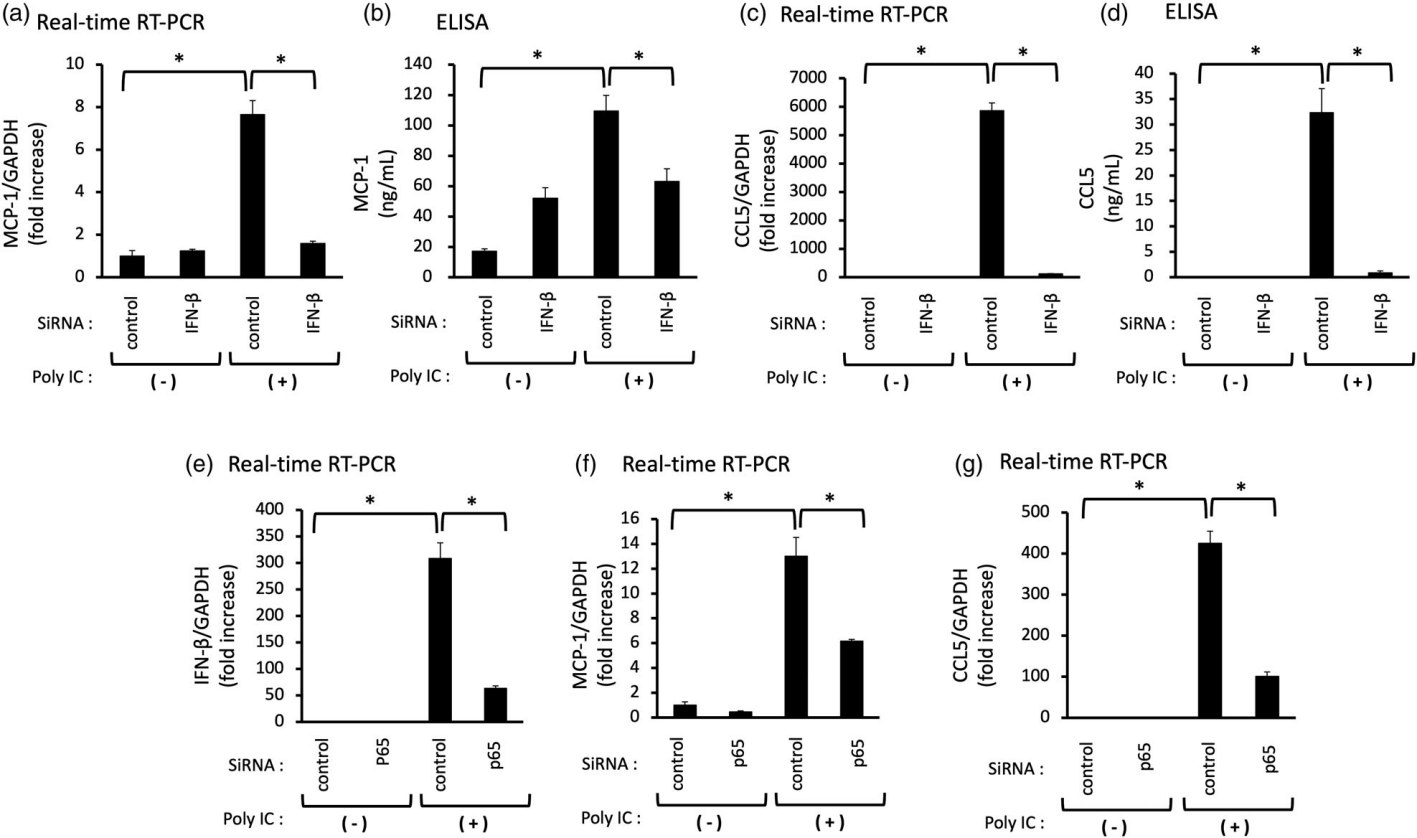
Knockdown of IFN-β decreases the poly IC-induced expression of both MCP-1 and CCL5, and knockdown of p65 decreases the poly IC-induced all of IFN-β, CCL5 and MCP-1 in cultured human GECs. The cells were transfected with siRNA against IFN-β, p65 or a non-silencing negative control siRNA. After 24h incubation, the cells were treated with 30 μg/mL poly IC for an additional 24 h (a–d, f, and g) or 2 h (e). The medium was collected, and RNA was extracted from cells, after which quantitative real-time RT-PCR analysis was performed. Data are shown as the means ± SD (*n* = 3, *p < 0.01 by *t*-test).

We examined the role of IFN-β and NF-κB in poly IC-induced MCP-1 and CCL5 expression. We performed RNA interference to knock down the expression of IFN-β or NF-κB p65．Knockdown of IFN-β or NF-kB p65 decreased the poly IC-induced the expression of MCP-1, CCL5 (Figure 3(a–d,f,g)), and knockdown of p65 decreased the expression of IFN-β mRNA (Figure 3(e)).

### CQ inhibits the expression of poly IC-induced MCP-1 and CCL5

**Figure 4.**
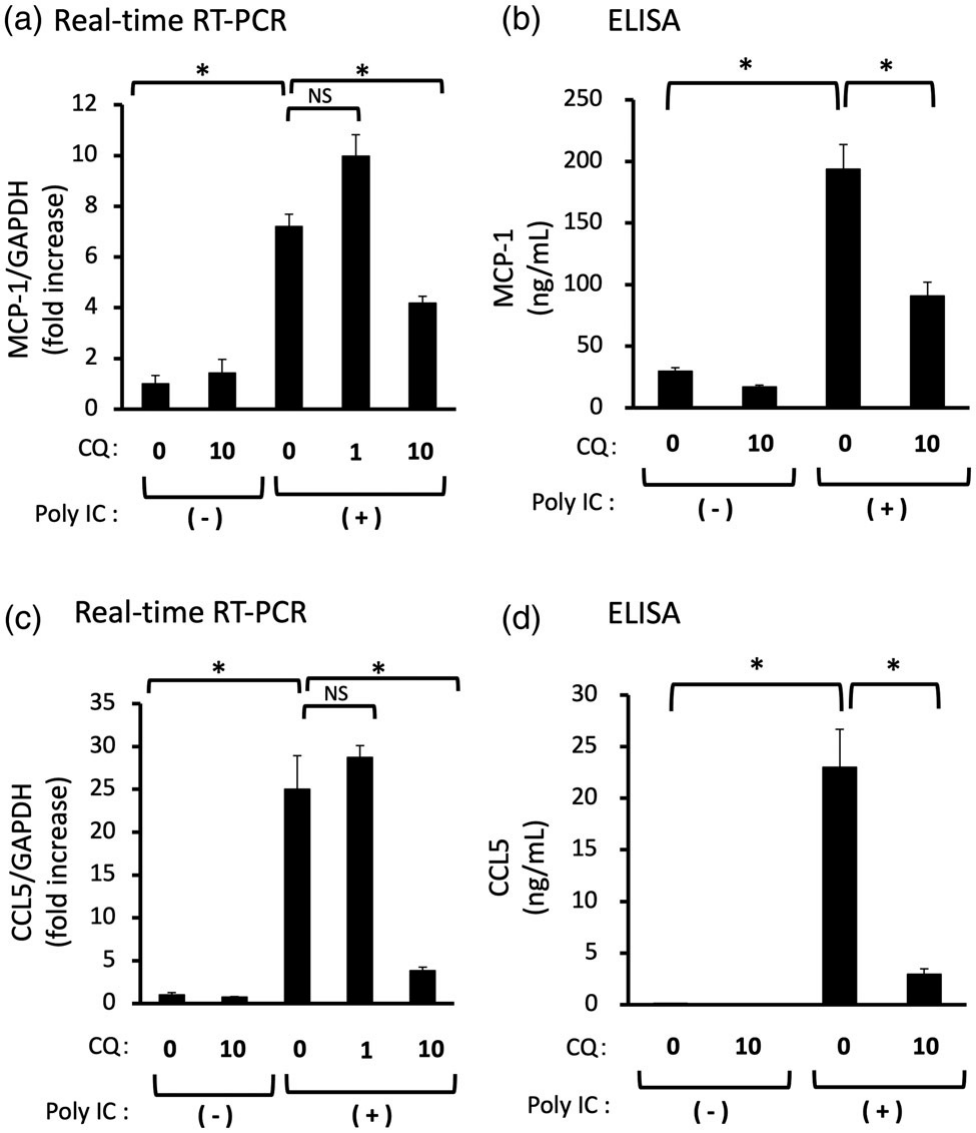
(a–d) Pretreatment with CQ inhibits the expression of both MCP-1 and CCL5 induced by poly IC. The cultured GECs were pretreated with 1 or 10 μg/mL CQ for 1 h and subsequently treated with 30 μg/mL poly IC for 16 h. The medium was collected, and RNA was extracted from cells, after which quantitative real-time RT-PCR and ELISA analyses were performed. Data are shown as the means ± SD (*n* = 3, *p < 0.01, by *t*-test).

We examined the effect of CQ on poly IC-induced MCP-1 and CCL5 expression. Pretreatment of cells with 1 μg/mL CQ did not inhibit the expression of MCP-1 and CCL5 mRNA, but 10 μg/mL CQ inhibited the poly IC-induced expression of MCP-1 and CCL5 mRNA and protein (Figure 4).

### HCQ inhibits the expression of poly IC-induced CCL5, but not MCP-1

**Figure 5.**
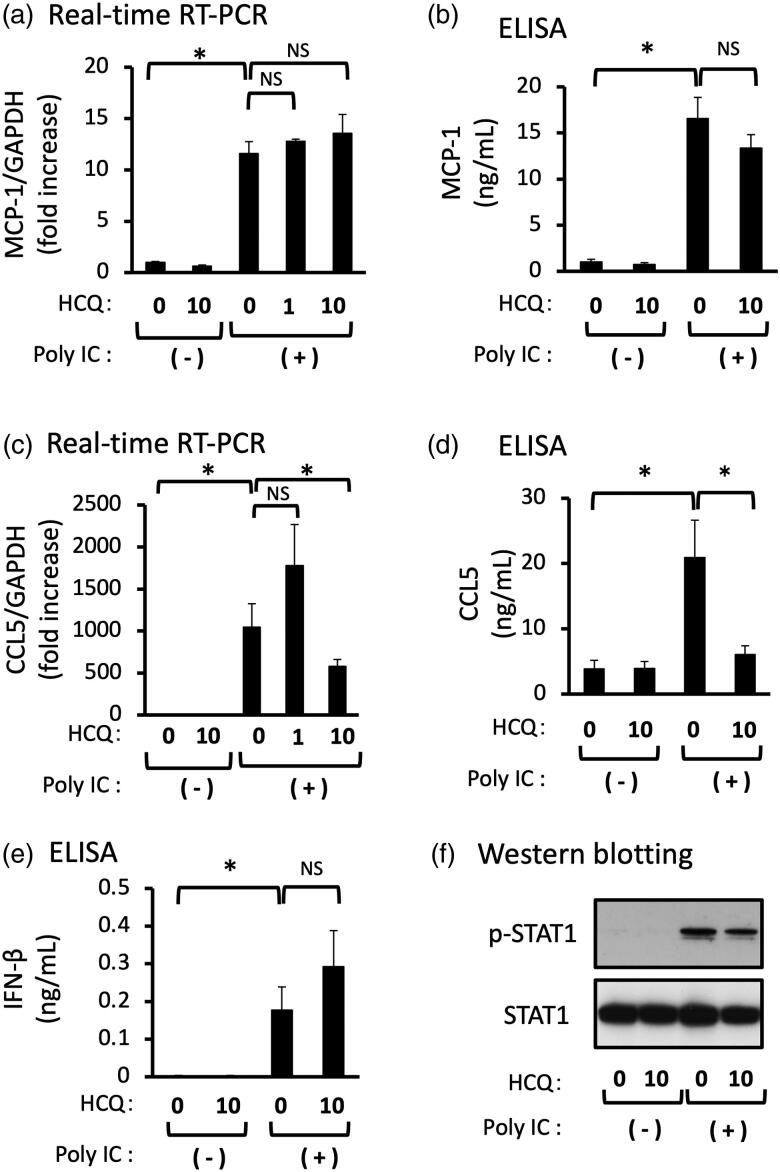
Pretreatment of GECs with HCQ inhibits the expression of CCL5, but not of MCP-1 and IFN-β. The cultured GECs were pretreated with 1 or 10 μg/mL HCQ for 1h and subsequently treated with 30 μg/mL poly IC for 16h. The medium was collected, and RNA was extracted from cells, after which quantitative real-time RT-PCR (a,c,e) and ELISA (b,d) analyses were performed. Data are shown as the means ± SD (*n* = 3, *p < 0.01, by *t*-test). (f) The cells were pretreated with 10 μg/mL HCQ for 1h, and then treated with 30 μg/mL poly IC for 6h. The cells were lysed and western blotting for phosphorylated STAT1 (p-STAT1) and STAT1 was performed.

We examined the effect of HCQ on the poly IC-induced expression of MCP-1, CCL5, and IFN-β. Pretreatment of cells with 1 μg/mL HCQ did not inhibit the mRNA expression of CCL5, but 10 μg/mL HCQ inhibited the mRNA and protein expression of CCL5 (Figure 5(c,d)). On the other hand, pretreatment of cells with 1 or 10 μg/mL HCQ did not inhibit the mRNA and protein expression of MCP-1 (Figure 5(a,b)). Poly IC-treatment induced expression of IFN-β protein and the phosphorylation of STAT1, but HCQ pretreatment did not result in a significant change of IFN-β protein and STAT1 phosphorylation (Figure 5(e,f)).

### DEX does not inhibit the expression of poly IC-induced either MCP-1 or CCL5

**Figure 6.**
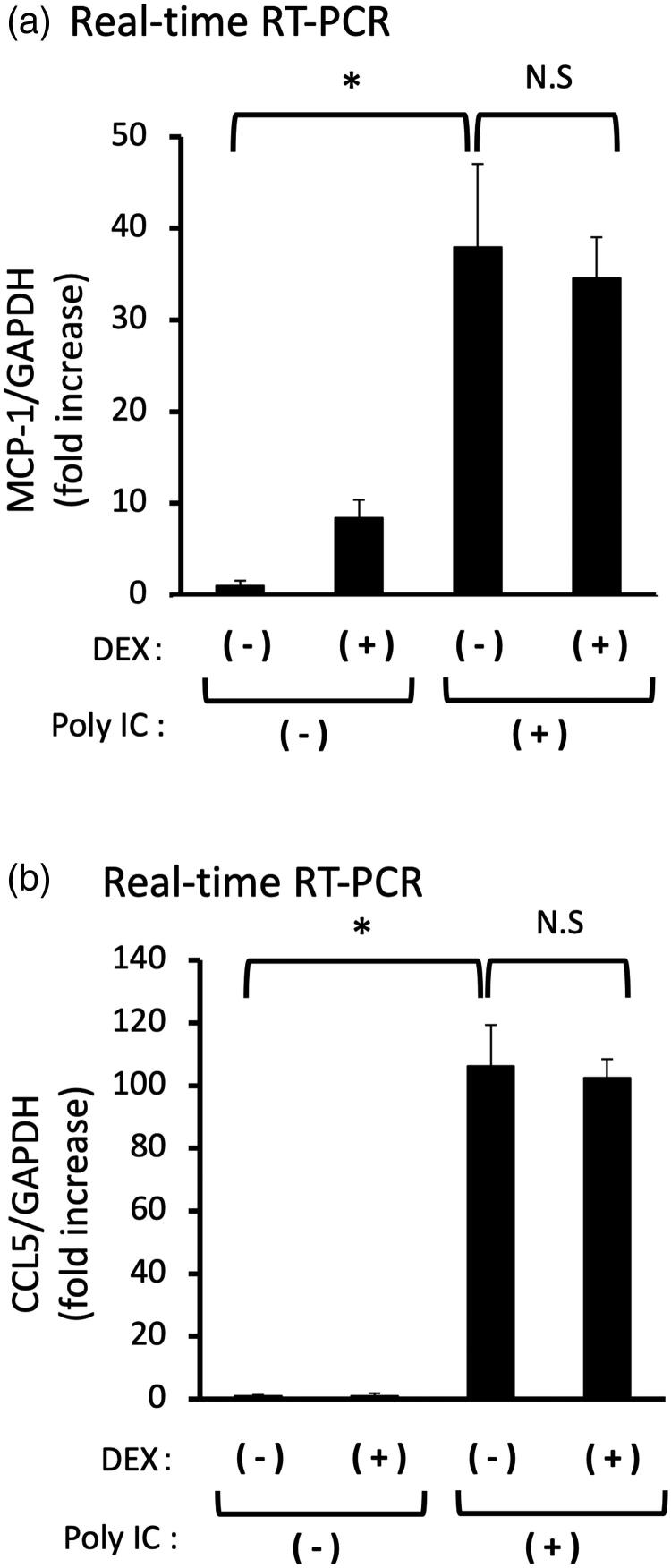
(a,b) Pretreatment of DEX does not inhibit the expression of both MCP-1 and CCL5 induced by poly IC. The cultured GECs were pretreatment with 10 μM DEX for 1h and subsequently treated with 30 μg/mL poly IC for 16h. The medium was collected, and RNA was extracted from cells, after which quantitative real-time RT-PCR analysis was performed. Data are shown as the means ± SD (*n* = 3, *p < 0.01, by *t*-test).

Pretreatment of cells with 10 μM DEX did not inhibit the expression of MCP-1 or CCL5 mRNA (Figure 6).

## Discussion

Since the activation of TLR3 signaling cascades results in the subsequent release of inflammatory chemokines, cytokines, adhesion molecules, and finally type I IFN [17], sustained activation of type I IFN via TLRs activation is thought to be involved in the pathogenesis of SLE [6]. Renal biopsy specimens showed apparently higher glomerular expression of TLR3 in patients with GN [7]. Thus, regional viral and ‘pseudo’ viral immunoreactions via the activation of TLR3/IFN-β signaling in the resident glomerular cells have been postulated to be involved, at least in part, in the pathogenesis of LN [3,4]. Recently, we found that the activation of TLR3/IFN-β axis induced endothelial expression of representative proinflammatory functional molecules, neutrophil chemoattractant C-X-C motif chemokine 1 (CXCL1)/GROα, macrophage chemoattractant CX3CL1/fractalkine, E-selectin, plasminogen activator inhibitor-1, and interleukin-6 (IL-6) in GECs [9–12]. Regarding type I IFNs, we confirmed that IFN-β, but not IFN-α, is released from MCs and GECs and acts as a mediator in resident renal cells [4,9–12]. We postulate that IFN-β is released from resident renal cells and acts as an ‘autocrine’ mediator, whereas IFN-α may be released from infiltrating proinflammatory cells and acts as a ‘paracrine’ mediator, although this theory remains to be elucidated [18]. Based on these results, we also speculated that despite some limitations, endothelial TLR3 signaling, which is associated with the continuous activation of type I IFN as well as a regional expression of various inflammatory molecules, is possibly involved in the pathogenesis of LN [3–5].

In the present study, we found that poly IC induced the expression of MCP-1 and CCL5 downstream of IFN-β in a time- and concentration-dependent manner in cultured human GECs. Notably, CQ effectively inhibited poly IC-induced both MCP-1 and CCL5 expression in GECs whereas HCQ inhibited CCL5 expression only. Since anti-inflammatory steroids are commonly used for the treatment of patients with LN, we next examined the inhibitory effect of DEX in this experiment. Interestingly, DEX did not inhibit the expression of MCP-1 and CCL5; thus, we speculated that DEX did not affect the TLR3/IFN-β axis in GECs. On the other hand, the anti-malarial agents, CQ and HCQ have some inhibitory roles in the TLR3/IFN-β axis in GECs, leading to decreased expression of MCP-1 and CCL5. In this context, the postulated mode of action of anti-malarial agents in TLR signaling is thought to be an inhibition of endosomal acidification [19]. On the other hand, it has been reported that anti-malarial agents do not influence the endosomal pH and expression of TLRs, suggesting that the anti-malarial agents interact with nucleic acids. These interactions consequently cause structural modification of the TLR ligands, which prevents their binding to TLR [20]. However, that may not be the case in the present study because anti-malarial agents have been added to the cells 1 h before poly IC treatment. Regarding TLR3 signaling in resident glomerular cells, our previous studies showed that CQ inhibits TLR3 signaling during the early phase of IFN-β production; that is, inhibition of nuclear translocation of phosphorylated nuclear factor-κB [12,15]. In this study, however, HCQ did not affect the poly IC induced-IFN-β protein expression in GECs (Figure 5(e)). Accordingly, HCQ may act downstream of IFN-β expression in GECs, suggesting that different modes of action on the endothelial TLR3/IFN-β signaling may exist between CQ and HCQ. However, we did not determine the detailed effect of HCQ on the TLR3 signaling because of some difficulties in experimental settings. Thus, it is imperative to conduct more detailed studies in the future.

A study reported a minimum HCQ target blood level >600 ng/mL to prevent flares in 171 lupus patients [21]. Considering the blood concentration level in clinical practice, 10 μg/mL of CQ and HCQ used in our experiments may be higher than that postulated in the abovementioned study. However, the adequate blood level of the drugs in patients with LN is yet to be determined. In addition, further studies should clarify whether adequate blood levels of the drugs can prevent activated TLR3 signaling in GECs. The limitation of this study is that we only examined in cell culture model *in vitro*. Further research of *in vivo* study may be needed to confirm our hypothesis when considering a clinical setting.

It has been reported that HCQ, a product obtained by adding a hydroxyl group to CQ, showed a marked decrease in the development of retinopathy. Among 647 patients treated with CQ for a mean period of more than 10 years, 16 (2.5%) patients developed definite retinal toxicity, whereas only 2 (0.1%) of 2043 patients who were treated with HCQ for a similar period developed retinopathy [16]. Based on the relationship between retinopathy and melanin, the affinity of melanin for HCQ is not as strong as that for CQ [22]. Further, HCQ was found to be a less potent enhancer of lipofuscinogenesis compared to CQ, apparently due to its less effective inhibition of lysosomal degradative capacity [23]. These different modes of action of CQ and HCQ may be attributable to their tendency to develop retinopathy, although this theory remains speculative. However, different modes of action between CQ and HCQ in resident glomerular cells remain unclear. To the best of our knowledge, this is the first study to show different modes of action that may exist between CQ and HCQ in TLR3 activation induced-proinflammatory chemokine expression in human GECs. In addition, these differences may be involved in the occurrence of adverse events of the drugs, although this theory remains speculative.

## Conclusion

Our results may further support the regional renoprotective effects of the anti-malarial agents, CQ, and HCQ in the development of inflammation in GECs, as postulated in LN [9,13,21]. Different modes of action between CQ and HCQ in TLR3 activation induced-proinflammatory chemokine expression in GECs may exist, which may also be involved in the occurrence of adverse drug reactions. However, more detailed studies are needed to confirm our preliminary findings.
